# Molecular characterization of *Chlamydia* species in commercial and backyard poultry farms in Costa Rica

**DOI:** 10.1017/S0950268821002715

**Published:** 2022-02-24

**Authors:** Antony Solorzano-Morales, Gaby Dolz

**Affiliations:** Universidad Nacional, Escuela de Medicina Veterinaria, Laboratorio de Investigación en Medicina Poblacional, P.O. Box 86-3000, Heredia, Costa Rica

**Keywords:** *Chlamydia gallinacea*, *Chlamydia muridarum*, *Chlamydia psittaci*, poultry

## Abstract

Outbreaks caused by *Chlamydia psittaci* and other chlamydial species have recently been reported in poultry farms worldwide, causing considerable economic losses. The objective of this study was to determine the presence of chlamydial species in these birds in Costa Rica. One hundred and fifty pools of lung tissue samples from industrial poultry with respiratory problems and 112 pools of tracheal swabs from asymptomatic backyard poultry were analysed by real-time quantitative polymerase chain reaction (qPCR), end-point PCR and sequencing. A total of 16.8% (44/262) samples were positive for *Chlamydia* spp., most of them detected in asymptomatic backyard poultry (28.6%, 32/112) and fewer in industrial poultry (8%, 12/150). Of these positive samples, 45.5% (20/44) were determined to be *C. psittaci*. For the first time *C. psittaci* genotype A is reported in poultry in Latin America. In addition, the presence of *Chlamydia gallinacea* in backyard poultry and of *Chlamydia muridarum* in industrial and backyard poultry is reported for the first time in Central America. In 40.9% (18/44) of the positive samples, it was not possible to identify the infecting chlamydial species. These findings reveal a zoonotic risk, particularly for poultry farm and slaughterhouse workers having direct contact with these birds.

## Introduction

Avian chlamydiosis or psittacosis is a zoonotic disease caused by the intracellular bacterium *Chlamydia psittaci*, which is widely distributed worldwide [1]. This bacterium can infect more than 467 species of birds and several species of mammals, including humans [2]. Its pathogenicity in infected birds depends on the affected species and the infecting *C. psittaci* strain.

*C. psittaci* strains are currently divided into 15 genotypes based on the sequence of the *ompA* gene, which encodes the major outer membrane protein [3, 4]. Most avian genotypes have also been sporadically identified in humans, especially genotypes A, B and EB [5, 6]. Transmission to humans occurs mainly through aerosols of faecal or respiratory secretions of birds. Worldwide, psittacosis is a notifiable disease in humans and companion birds, and it has recently become so in poultry [7]. Although chickens and turkeys initially seemed to be less susceptible to chlamydial infection and to be a sporadic source of human infection [8], studies often reported *C. psittaci* in this type of birds and its transmission to humans [9, 10]. However, since the discovery of *Chlamydia gallinacea* this species seems predominant, either exclusively or in conjunction with *C. psittaci* in chicken flocks [7, 11–13]. Also other chlamydial species have been reported in poultry, including *Chlamydia abortus*, *Chlamydia pecorum*, *Chlamydia trachomatis*, *Chlamydia suis* and *Chlamydia muridarum* [14]. Recent studies hypothesised that *C. gallinacea* is endemic in chickens and causes mild clinical signs and reduced body weight gain in broilers [14]. The zoonotic potential of *C. gallinacea* has been suggested, but no conclusive evidence has been presented to date [7].

Studies conducted in Costa Rica identified the presence of *C. psittaci* in psittacines and pigeons cohabitating with Costa Ricans in homes and public places, respectively [15, 16]. However, the presence of *C. psittaci* and other *Chlamydia* species in gallinaceous birds is unknown. The present study aimed to determine the presence of chlamydial species in industrial and backyard poultry of Costa Rica, which could be transmitted to humans during bird handling and slaughter.

## Materials and methods

### Reference population and study type

In Costa Rica, industrial commercial farms are establishments with a veterinary certificate of operation issued by the National Animal Health Service (Servicio Nacional de Salud Animal – SENASA) and characterised by having more than 100 birds. The backyard farms are facilities with a maximum of 100 birds in confinement or in freedom, used for subsistence purposes in a non-organised or technical way. The main broiler genetic lines used in the country are Cobb 500, Ross 308 and Hubbart, and the main egg layers are Isa Brown, Hy-line Brown and Lohman (R. Chaves, National Avian Health Program – SENASA Coordinator, pers. com.). A study with non-probabilistic convenience sampling was conducted to determine the presence of *Chlamydia* species in gallinaceous birds with and without respiratory symptoms. Two groups of samples were analysed, which were collected in 2014 and 2015 by the SENASA. The first group (group 1) of samples consisted of lung tissues collected from broiler chickens (*Gallus gallus domesticus*) with respiratory problems, from industrial–commercial systems in the central region (Alajuela, Cartago, Heredia and San José) and Puntarenas. A total of 150 pools of lung tissue samples from birds of 77 industrial–commercial production establishments were analysed. Each pooled sample consisted of lung tissues from one to five chickens with respiratory symptoms from the same production farm. The second group (group 2) consisted of tracheal swabs taken from chickens and turkeys (*Meleagris gallopavo*) without respiratory signs, from backyard farms in different geographical areas of the country (Alajuela, Cartago, Guanacaste, Heredia and Puntarenas). In this group a total of 112 pools of tracheal swab samples from birds without clinical signs from 25 backyard poultry establishments were analysed; 111 pools of samples were swabs from one to five chickens, and one pool of samples was swabs from three turkeys. The samples were kept cold in an ice chest for a maximum of 24 h until being sent to the laboratory, where they were immediately preserved at −80 °C.

### DNA extraction from bird samples

For nucleic acid extraction, the MagMAX™ Pathogen RNA/DNA Kit (Life Technologies, Carlsbad, CA, USA) and the MagMAX™ Express-96 Magnetic Particle Processor (Applied Biosystems, Foster City, CA, USA) were used following the manufacturer's instructions. For the lung tissues bead-beating method was used as a preparative step prior to DNA extraction. A NanoDrop^®^ ND-1000 spectrophotometer was used to quantify and verify the quality of the extracts.

### Quantitative polymerase chain reaction (qPCR) to detect *Chlamydia* spp.

The qPCR followed the protocol described by Everett *et al*. [17] for the detection of the 23S rRNA gene of the family Chlamydiaceae, with the following modifications: the primers were TQF 5′-GAAAAGAACCCTTGTTAAGGGAG-3′ and TQR 5′-CTTAACTCCCTGGCTCATCATG-3′, and the probe was FAM-CAAAAGGCACGCCGTCAAC-TAMRA. The reaction volume (25 μl) included 12.5 μl of Maxima Probe/ROX qPCR Master Mix – 2× (Thermo Scientific, Waltham, MA, USA), 1.0 μl of each primer at 10 pmol/μl, 0.5 μl of the probe at concentrations 10 pmol/μl, 5 μl of DNA and 5 μl of molecular biology-grade water (Thermo Scientific). The amplification steps were 95 °C for 10 min followed by 40 cycles of 95 °C for 15 s and 60 °C for 1 min. The poultry samples were analysed in triplicate. A DNA extract from *C. muridarum* (ATCC VR-123), donated by the Laboratory of Chlamydias and Human Papillomavirus, Virology Institute, School of Medical Sciences, National University of Córdoba, Argentina was used as a positive control and molecular biology-grade water as a negative control. All samples with amplification of the 130-bp segment and with a growth curve exceeding the cycle threshold (automatically calculated) up to cycle 35 were considered positive [17].

### qPCR to detect *C. psittaci* in samples qPCR positive for *Chlamydia* spp.

The *C. psittaci ompA* gene amplification protocol described by Pantchev *et al*. [18] was implemented, with following modifications: the primers used were CppsOMP1-F (5′-CACTATGTGGGAAGGTGCTTCA-3′) and CppsOMP1-R (5′-CTGCGCGGATGCTAATGG-3′), and the probe was CppsOMP1-S (5′-FAM-CGCTACTTGGTGTGAC-TAMRA-3′). The reagent volumes and amplification conditions were the same as described above. The positive control was a *C. psittaci* DNA extract donated by the Laboratory of Chlamydia's and Human Papillomavirus, Argentina. All samples with amplification of a 77-bp segment up to cycle 36 were considered positive [18].

### Molecular characterisation and comparative phylogenetic analysis of samples positive for *Chlamydia* spp.

The samples that were positive in the qPCR for *Chlamydia* spp. were subjected to conventional PCR to amplify a partial sequence of the variable domain of the 23S rRNA gene of *Chlamydia* spp. [14]. The reaction mix (25 μl) included 12.5 μl of DreamTaq™ PCR Master Mix – 2× (Thermo Scientific, Waltham, MA, USA), 2.0 μl of each primer (23S-UP: 5′-GAGTCCGGGAGATAGACAGC-3′; 23S-DN: 5′-CATGGATCTTCACTAGTATCCGC-3′) at 10 pmol/μl, 5 μl of DNA and 3.5 μl of molecular biology-grade water (Thermo Scientific). The amplification steps consisted of 95 °C for 3 min; 40 cycles of 95 °C for 30 s, 50 °C for 30 s and 72 °C for 45 s; and a final extension at 72 °C for 5 min. The positive control used was the *C. psittaci* DNA extract mentioned above. Samples with amplicons of 329 bp were considered positive. PCR products were purified with the QIAquick^®^ kit (QIAGEN, Venlo, Netherlands) following the manufacturer's instructions, and were sent to Macrogen (Seoul, Korea) for sequencing. The partial sequences were aligned with the BioEdit Sequence Alignment Editor^®^ program [19] and were compared using the BLASTn algorithm against the NCBI database. Then the sequences were imported into MEGA X, where the Jukes and Cantor algorithm [20] and neighbour-joining method [21] were used to draw a phylogenetic tree. Sequences of reference strains of different chlamydial species (*C. gallinacea* 08-1274/3 (AWUS01000004), *Chlamydia avium* 10DC88 NR121988, *C. abortus* S26/3 (NR077001), *C. psittaci* 6BC (NR102574), *Chlamydia felis* Fe/C-56 (NR076260), *Chlamydia caviae* GPIC (NR076195), *Chlamydia pneumoniae* CWL029 (NR076161), *C. pecorum* E58 (NR103180), *C. suis* R22 (U68420), *C. trachomatis* 434/BU (NR103960) and *C. muridarum* Nigg3 (CP009760)) were included in the phylogenetic analysis.

### Genotyping and phylogenetic analysis of samples positive for *C. psittaci*

On the samples that were positive in the qPCR for *C. psittaci*, nested PCR was performed to amplify the variable domain IV of *ompA* gene and determine the genotype present. The protocol described by Sachse and Hotzel [22] was followed. The primers 191CHOMP (5′-GCIYTITGGGARTGYGGITGYGCIAC-3′) and 371CHOMP (5′-TTAGAAICKGAATTGIGCRTTIAYGTGIGCIGC-3′) were used in the first amplification round, and the pair 218PSITT (5′-GTAATTTCIAGCCCAGCACAATTYGTG-3′) and 336CHOMP (5′-CCRCAAGMTTTTCTRGAYTTCAWYTTGTTRAT-3′) in the second round. In both PCR runs, the reaction volume (25 μl) included 12.5 μl of DreamTaq™ PCR Master Mix – 2× (Thermo Scientific, Waltham, MA, USA), 1.0 μl of each primer at 20 pmol/μl, 5 μl of DNA and 5.5 μl of molecular biology-grade water (Thermo Scientific). The amplification steps were 95 °C for 3 min; 35 cycles of 95 °C for 30 s, 55 °C for 30 s and 72 °C for 45 s; and a final extension at 72 °C for 5 min. Samples with amplification of a 389-bp segment were considered positive. The amplicons were visualised by electrophoresis, purified and sent for sequencing to Macrogen (Korea).

Phylogenetic analysis was performed with the use of the genotype reference sequences A (AY762608), B (AF269265), C (L25436), D (AF269266), E (X12647), F (AF269259), E/B (AY762613), M56 (AF269268) and WC (AF269269) [23]. The tree was based on comparisons with the *ompA* sequence of *C*. *caviae* as an external group (GPIC, GenBank AF269282) [24].

## Results

A total of 44 (16.8%) of 262 samples were positive for *Chlamydia* spp. by the family-specific qPCR, 12 (8.0%) in group 1 and 32 (28.6%) in group 2 (Table 1). The analysis of the 44 positive samples by the conventional PCR for *Chlamydia* spp. established a chlamydial species in eight samples (Table 1). The qPCR for *C. psittaci* identified 20 positive samples: four (4/44, 9.1%) in group 1 and 16 (16/44, 36.3%) in group 2. Of the 20 positive samples, three were confirmed as *C. psittaci* by specific-nested PCR (Table 1). It was not possible to identify the chlamydial species in the remaining 18 samples with the end-point PCR.

**Table 1.** Numbers of positive samples amplified with different PCR techniques by production system

Production system	qPCR *Chlamydia* spp. +/total (%)	PCR *Chlamydia* spp. +/total (%)	qPCR *C. psittaci* +/total (%)	PCR *C. psittaci* +/total (%)	Unidentified species +/total (%)
Industrial (group 1)	12/150 (8.0)	2/12 (16.7)	4/12 (33.3)	2/4 (50.0)	7/12 (58.3)
Backyard (group 2)	32/112 (28.6)	6/32 (18.7)	16/32 (50.0)	1/16 (6.2)	11/32 (34.4)
Total	44/262 (16.8)	8/44 (18.2)	20/44 (45.5)	3/20 (15.0)	18/44 (40.9)

**Fig. 1.**
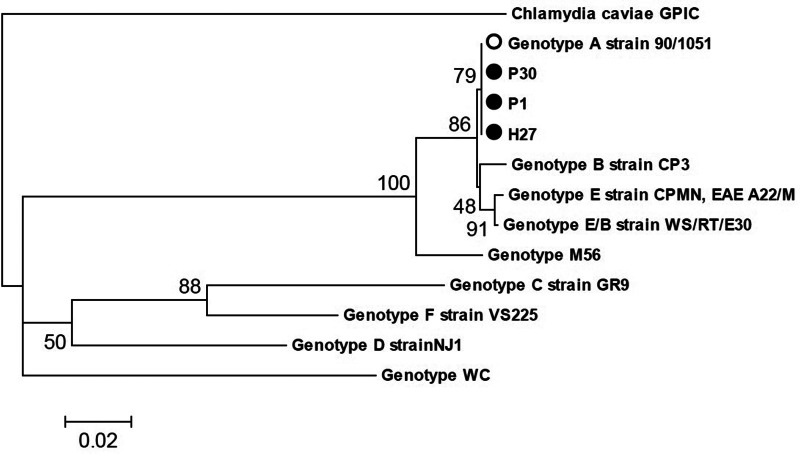
Dendrogram obtained from a 330-nucleotide fragment of variable domain IV of the *ompA* gene of *C. psittaci*, constructed using the neighbour-joining method and the Jukes and Cantor model. The sequence of *C. caviae* GPIC was included as an external group. The bootstrap values (10 000 pseudoreplicates) are indicated at the branch nodes.

**Fig. 2.**
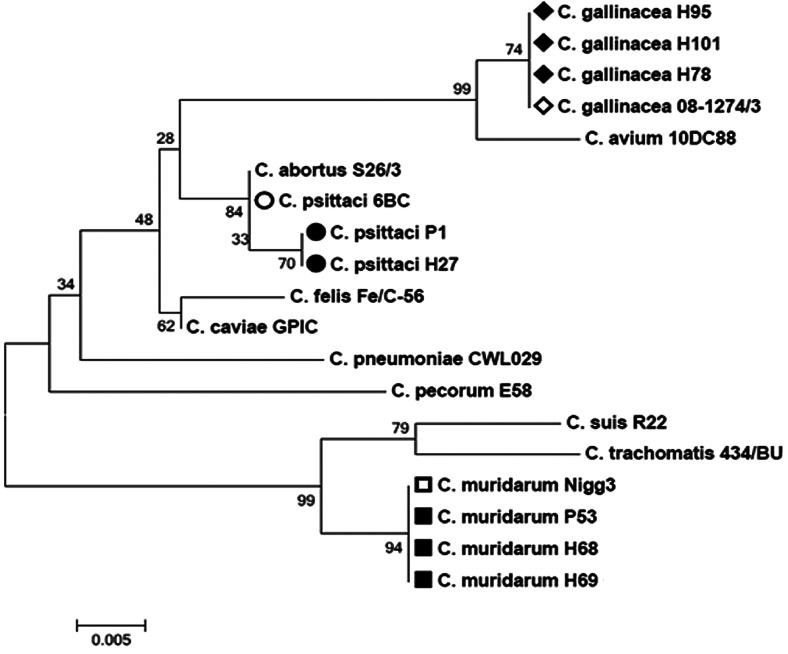
Dendrogram obtained from a 317-nucleotide fragment of the variable domain of the 23S rRNA gene of *Chlamydia* spp., constructed using the neighbour-joining method and the Jukes and Cantor model. Eleven reference strains of *Chlamydia* spp. and the chlamydial species found in the present study (bolded bullet points) are shown. The bootstrap values (10 000 pseudoreplicates) are indicated at the branch nodes.

Of the 12 positive samples in group 1, four samples were determined to be *C. psittaci* by qPCR. Of these, two samples (P1 and P30) were also confirmed by species-specific PCR and sequencing (Table 2, Fig. 1). One of these samples (P1) was also positive in the PCR for *Chlamydia* spp. and was sequenced as *C. psittaci* (Table 3, Fig. 2), while another sample (P53) was sequenced as *C. muridarum* (Table 3, Fig. 2). It was not possible to establish the infecting chlamydial species in seven samples (Table 1).

**Table 2.** Samples positive for the *ompA* gene of *C. psittaci* according to bird species, production system, location and nucleotide identity with GenBank sequences

Sample code	Bird species	Production system	Province	Nucleotide similarity (bp)	Standard strain (GenBank code)	Accession no.
P1	*G. gallus*	Industrial	Alajuela	100% (330/330)	*C. psittaci* (X56980)	OM327388
P30	*G. gallus*	Industrial	Alajuela	100% (330/330)	*C. psittaci* (X56980)	OM327389
H27	*M. gallopavo*	Backyard	Puntarenas	100% (330/330)	*C. psittaci* (X56980)	OM327390

**Table 3.** Samples positive for *Chlamydia* spp. according to bird species, production system, location and the nucleotide identity of the PCR-amplified 23S rRNA gene with GenBank sequences

Sample code	Bird species	Production system	Province	Nucleotide similarity (bp)	Standard strain (GenBank code)	Accession no.
P1	*G. gallus*	Industrial	Alajuela	99.7% (316/317)	*C. psittaci* (NR102574.1)	OM758106
P53	*G. gallus*	Industrial	Alajuela	99.4% (327/329)	*C. muridarum* (CP007217.1)	OM758211
H27	*M. gallopavo*	Backyard	Puntarenas	99.4% (323/325)	*C. psittaci* (NR102574.1)	OM758117
H58	*G. gallus*	Backyard	Alajuela	99.4% (327/329)	*C. muridarum* (CP007217.1)	OM793056
H59	*G. gallus*	Backyard	Alajuela	99.4% (327/329)	*C. muridarum* (CP007217.1)	OM793056
H102	*G. gallus*	Backyard	Puntarenas	100% (327/327)	*C. gallinacea* (MK294049.1)	OM760045
H105	*G. gallus*	Backyard	Puntarenas	100% (327/327)	*C. gallinacea* (MK294049.1)	OM793052
H112	*G. gallus*	Backyard	Cartago	100% (327/327)	*C. gallinacea* (MK294049.1)	OM793056

Of the 32 samples positive for *Chlamydia* spp. in group 2, 16 samples were determined to be positive for *C. psittaci* by qPCR and one of these samples (H27) was confirmed by the two end-point PCR assays and sequencing (Tables 2 and 3, Figs 1 and 2). Using conventional PCR for *Chlamydia* spp., three samples (H102, H105 and H112) were determined to be positive for *C. gallinacea* (Table 3, Fig. 2), and two samples (H58 and H59) were determined to be positive for *C. muridarum* (Table 3, Fig. 2). It was not possible to determine the chlamydial species in 11 samples (Table 1).

The three samples positive for *C. psittaci* were established as genotype A and received the GenBank accession numbers listed in Table 2. The phylogenetic analysis based on the 23S rRNA gene showed that the sequences of the three species identified in this study (*C psittaci*, *C. muridarum* and *C. gallinacea*) were similar (99.4–100%) to sequences of chlamydial species deposited in GenBank (Table 3) and received the accession numbers listed in Table 3.

The greatest number of positive *Chlamydia* samples was found in the group of backyard birds without clinical signs (group 2). A total of 28.6% (32/112) of these samples were positive. Positive samples were found in 60.0% (15/25) of the analysed backyard establishments (Table 4). In contrast, in the group of samples from birds from industrial–commercial establishments, only 8.0% (12/150) were positive, and positive birds were found in 12.9% (10/77) of the analysed establishments. Samples positive for *Chlamydia* spp. were found mainly in Alajuela (33/44, 75.0%), though the largest number of samples was also collected in this province (195/262, 74.4%). All samples positive for *Chlamydia* spp. from group 1 (industrial–commercial birds) were found in the province of Alajuela (Table 4), while positive samples from group 2 (backyard birds) were found mainly in Alajuela but also in Puntarenas, Cartago and Guanacaste (Table 4).

**Table 4.** Distribution of samples positive for *Chlamydia* spp. by production system and location

Production system	Province	Number of samples (%)	+qPCR (%)	Establishments analysed (+)	Animals analysed
Industrial (group 1)	Alajuela	133 (88.7)	12 (8.0)	65 (10)	534
	Cartago	6 (4.0)	0	4 (0)	22
	Heredia	4 (2.7)	0	3 (0)	18
	San José	4 (2.7)	0	3 (0)	20
	Puntarenas	3 (2.0)	0	2 (0)	11
	Total	150 (100.0)	12 (8.0)	77 (10)	605
Backyard (group 2)	Alajuela	62 (55.4)	21 (18.7)	13 (10)	310
	Cartago	6 (5.4)	1 (0.9)	3 (1)	30
	Guanacaste	6 (5.4)	1 (0.9)	1 (1)	30
	Heredia	12 (10.7)	0	2 (0)	60
	Puntarenas	26 (23.2)	9 (8.0)	6 (3)	125
	Total	112 (100.0)	32 (28.6)	25 (15)	555
Total		262	44	102 (25)	1160

The presence of *C. psittaci* was detected in three commercial establishments in the province of Alajuela, four backyard establishments in Alajuela and one backyard establishment in Puntarenas (*C. psittaci*, qPCR) (Table 2), while *C. gallinacea* was detected in two backyard establishments in Puntarenas and one in Cartago (Table 3). The presence of *C. muridarum* was detected in one commercial establishment and in one backyard establishment in Alajuela (Table 3).

## Discussion and conclusion

The present study reports the first detection of different *Chlamydia* species in gallinaceous birds from commercial and backyard farms in Costa Rica and in Central America. The percentage of positivity observed in pooled samples collected from commercial farms (8.0%) was higher than that reported in Mexico (3.4% (20/526 individual samples)) and Slovakia (6.9% (19/276 individual samples) [9, 13] but lower than that found in commercial poultry farms in Poland (23% (26/113 pooled samples)), Netherlands (49% (74/151 pooled samples)) and Argentina (40.3% (27/67 individual samples)) [7, 25, 26]. Likewise, the percentage of positivity detected in the backyard farms in Costa Rica (28.6%) was higher than that in other countries, such as the United States (13.6% (64/472 pooled samples)), Italy (15% (24/160 individual samples)) and China (24.7% (442/1791 individual samples)) [11, 12, 14], but similar to that recently reported in Mexico (28.6% (83/293 individual samples)) [13].

Four times as many positive samples for *Chlamydia* species were found in samples from backyard birds as from industrial birds, although the latter were the ones showing respiratory signs. This finding may be due to the lack of biosecurity measures in backyard establishments and the higher likelihood of contact with other animals, mainly wild birds that can transmit the agent [27]. Factors such as strict biosecurity measures, good cleaning and disinfection practices, use of preventive medicine (antibiotics) and good nutritional management have been shown to reduce the risk of pathogen transmission [28]. The detection of *C. psittaci* in birds with respiratory signs in commercial farms is noteworthy. However, the positivity percentages in the two groups may be underestimated because, on the one hand, the bacterium is excreted intermittently in asymptomatic animals [29] and, on the other hand, birds typically excrete the bacterium through either the pharynx or cloaca and not from both sites [9]. Zoonotic potential should be assumed possible in asymptomatic carriers of *C. psittaci* even without the presence of clinical signs [30].

This study is the first to detect in Costa Rica and in Central America the presence of *C. psittaci* in commercial and backyard gallinaceous birds. Its presence should alert poultry farm and slaughterhouse workers and others who have direct contact with these birds that they might be at risk of infection by the bacterium and the illness it can cause. *C. psittaci* has been detected in chickens in Australia, Germany, Belgium, France, Slovakia, Italy and China [9, 14], causing economic losses to the poultry industry due to its mandatory reporting [9]. The percentages of infection in all these countries do not exceed 6.9% (Slovakia), in contrast to the percentage obtained in the present study (45.5%). However, serological studies (enzyme-linked immunoassay specific for *C. psittaci*) in fattening farms in Belgium found 95% seropositivity, so the percentages obtained by PCR could be underestimated due to the intermittent excretion of the bacterium [31]. The detection of *C. psittaci* genotype A in poultry in our country agrees with reports from Belgium [8] and represents a risk for people who have contact with these birds, since this genotype is considered highly virulent [32]. In the different establishments, especially those that were positive for *C. psittaci*, personal protection measures should be reviewed and implemented, which should include a hand-hygiene protocol and protective clothing, including gloves and full-face air-filter masks. Also, there must be a transition room where protective clothing can be stored, as well as adequate cleaning and natural or mechanical ventilation to avoid cross-contamination between the different spaces [33].

The diagnosis of infectious agents that cause respiratory problems in poultry in Costa Rica is actively carried out by SENASA, which efforts cover Newcastle disease, avian influenza, avian infectious laryngotracheitis, avian infectious bronchitis and infection by *Mycoplasma* species. A recent study by De Boek *et al*. [34] found problems of conjunctivitis, upper respiratory disease and dyspnoea in broilers, and established that *C. psittaci* always preceded an *Ornithobacterium rhinotracheale* infection, providing evidence that *C. psittaci* could occur at an early age in broilers without a predisposing respiratory infection. Also increasing mortality of avian influenza virus H9N2 by suppressing host immune responses was reported due to infections with pathogenic *C. psittaci* strains [35]. Therefore, it is recommended to include avian chlamydiosis in the differential diagnosis of respiratory diseases of poultry [8, 31].

The presence of *C. muridarum* in commercial and backyard chickens is also reported here for the first time in Costa Rica and in Central America. This finding is considered accidental and sporadic, possibly due to close contact of the birds with its natural hosts (rodents) [14].

Finally, the presence of *C. gallinacea* in backyard chickens is reported for the first time in Costa Rica and in Central America. This chlamydial agent has recently been detected worldwide, so little information is available on it. In America, its presence is reported in Argentina, Mexico and the United States [11, 13, 26]. Experimental studies with *C. gallinacea* have shown a significant reduction in body weight (6.5–11.4%) in animals without clinical signs [14].

The qPCR techniques used in this study were more sensitive than end-point PCR techniques, as widely documented in the literature [1]. Of the samples detected as positive in the qPCR for *Chlamydia* spp., 45.4% (20/44) were confirmed as *C. psittaci* by the qPCR specific to this species, confirming the significant presence of the agent in our environment [15, 16]. The end-point PCR assays for *Chlamydia* spp. and *C. psittaci*, in contrast, were only able to detect 18.2% (8/44) and 15.0% (3/20) of the positive samples detected by qPCR, respectively. In 40.9% (18/44) of the positive cases, it was not possible to identify the infecting chlamydial species. It is possible that the use of qPCR for other *Chlamydia* species (e.g. *C. gallinacea*) could help us to identify the undetermined chlamydial species. Recent studies [9, 14] have established that the majority of unidentified chlamydia's belong to *C. gallinacea* (detected by qPCR), a species considered endemic and predominant in chickens.

The results of this study demonstrate the complexity of the epidemiology of avian chlamydiosis and confirm that chlamydial infections in birds are not only due to *C. psittaci*. The results were reported to the director and the officials of SENASA, to veterinary professionals and students, through press releases and congresses. We recommend alerting individuals who work in commercial poultry farms or have contact with birds about the risk of contagion with chlamydial agents so that they can take the necessary biosecurity measures. In addition, it is necessary to raise awareness among veterinary professionals and remind them to consider chlamydia's in the differential diagnosis of agents causing respiratory problems in poultry. Finally, SENASA should include the diagnosis of avian chlamydiosis in its active control of respiratory diseases in poultry to avoid the spread of infection. Molecular diagnostic methods, especially qPCR, thanks to their high sensitivity and specificity, should be the first choice to determine the presence of *Chlamydia* species in poultry. Future studies should investigate the pathogenicity, effect on production and possible zoonotic potential of *C. psittaci* and *C. gallinacea* in poultry of Costa Rica.

## Data Availability

The data that support the findings of this study are available on request from the corresponding author. The data are not publicly available due to restrictions apply to the availability of these data, which were used under license for the current study, and so are not publicly available. Data are however available from the authors upon reasonable request and with permission of SENASA, Costa Rica.
